# Reflections on the Collaborative Fight Against COVID-19

**DOI:** 10.3389/fmed.2020.00565

**Published:** 2020-09-17

**Authors:** Michel Goldman, Mitchell Silva

**Affiliations:** ^1^Institute for Interdisciplinary Innovation in Healthcare (I3h), Université Libre de Bruxelles, Brussels, Belgium; ^2^EUPATI Belgium vzw, Brussels, Belgium

**Keywords:** patient, trial, partnership, regulation, COVID-19

## Abstract

Clinical trials to identify efficient treatments against COVID-19 flourish worldwide without much attention to patients' voice so far. As therapeutic interventions in the early phase of the disease are attracting more and more interest, we argue that now is the time to involve patients' organizations in the design of clinical protocols in order to define the most relevant end-points and assess the risk-benefit balance of new therapies.

Since the end of December 2019 when a pneumonia outbreak of unknown origin was reported in China, scientific knowledge on COVID-19 progressed at an unprecedented pace. Within a few months, SARS-CoV-2 was identified as the culprit agent, mechanisms by which it infects cells were deciphered and causes of organ damage were identified. This has been possible because scientific data were never shared so quickly and transparently, with essential results being exchanged and made available in real time to the entire scientific community. Now that we enter a new phase during which science must be translated in innovative medicines and vaccines, a key question is whether collective intelligence will remain mobilized in a similar manner.

Indeed, collaboration across borders will be essential to overcome COVID-19. Not only collaboration between countries, but also between academia and industry, and between public and private sectors. The challenge is not only to discover the right targets for anti-COVID-19 therapies and vaccines, but also to ensure that the new products will be both efficient and safe, that they can be manufactured at full industrial scale, and that they will be accessible worldwide at affordable prices. Several initiatives have been launched to meet this challenge and provide incentives to foster collaborative endeavors. Both the “Accelerating COVID-19 Therapeutic Interventions and Vaccines” (ACTIV) initiative in the USA (1) and the “Coronavirus Global Response” launched by the European Commission (https://global-response.europa.eu/index_en) will support public-private partnerships and multi-stakeholder consortia. In this editorial perspective, we argue that patients' voice will be essential to ensure uptake in the wider public of new therapies and vaccines resulting from these initiatives.

## COVID-19 Clinical Studies So Far

During the exploding phase of the pandemic, the immediate priority was to identify compounds able to mitigate the most severe cases of COVID-19. Indeed, doctors facing patients dying of fulminant pneumonia desperately look for potential therapies that can be immediately available. Unfortunately, the overall outcome of the clinical trials conducted during the initial phase of the pandemic was by and large disappointing. A huge number of small statistically underpowered trials were simultaneously launched (2) and a recent paper reported that only 30 out of 1,840 registered trials have actually been reported, and very few trials addressed early interventions aiming at preventing hospitalization (3). The most pertinent trials were the so-called adaptive trials which conciliate the scientific rigor brought by randomization and the ethical duty to provide patients with a potentially beneficial therapy. Indeed, adaptive trial designs allow to change therapeutic options in the course of a study according to interim evaluations and inefficient treatments can be withdrawn whereas newly emerging therapies can be introduced in the trial (4). Three large trials using such an adaptive design were launched, *Solidarity* led by the World Health organization (WHO), *Discovery* by the French Institute for Health and Medical Research (INSERM), and *Recovery* by the University of Oxford. Unfortunately, the implementation of *Solidarity* and *Discovery* were hampered by regulatory and administrative hurdles. On the other hand, *Recovery* provided an essential information for the management of future cases by demonstrating the beneficial action of dexamethasone in reducing the mortality of patients with the most severe forms of the disease (5). Remdesivir is the other treatment which has been scientifically validated in a randomized study, again in hospitalized patients with serious illness (6).

## How Should Future Trials Look Like?

While a number of countries are still in the ascending limb of the pandemic and several others face resurgence of new cases, now is the time to reflect on how to make the search for new COVID-19 therapies more efficient (7). Fore and foremost, radical changes are necessary to prevent a detrimental competition between trials that are either underpowered or unrealistic in terms of patient accrual. This is an ethical duty toward patients which agree to be exposed to potential risks with the understanding that by doing so they contribute to create firm evidence regarding efficacy and safety of the new medicines they receive. Therefore, it is essential that trials are designed to include sufficient numbers of patients within the period of time during which the pandemic is expected to be active. The insufficient pace of patient enrollment is indeed the most frequent cause of trial failure. This has been a matter of great concern during the recent Ebola virus and Zika virus outbreak (8). More than ever, there is a huge need for better coordination and management of trials through the establishment of collaborative infrastructures and platforms. In this regard, besides the ACTIV program in the US (https://bit.ly/32Ct8Fz), the Accelerating COVID-19 Research and Development platform (ACCORD) in the UK (https://bit.ly/30vzhRj) and the ERAvsCORONA action plan in the European Union (https://bit.ly/2CBKrfb) are highly welcome initiatives. Regulators have also an important role in facilitating the implementation of sound clinical trials for COVID-19. Fortunately, European and US regulatory agencies have issued guidelines for clinical investigations during pandemics without compromising on their scientific validity and ethical conduct (8). Finally, as discussed in the next section, patients should now be invited to take an active role in the design of COVID-19 trials.

## Empowering Patients

So far, patients enrolled in the trials mentioned hereabove did not have much other option than signing the informed-consent form to get a chance to receive a potentially life-saving medicine. As more and more patients will be diagnosed at an earlier stage of the disease or with a milder form of COVID-19, investigators will have to ensure that patients fully understand and adhere to the rationale of the proposed trials, their expected benefits and their potential risks.

Patients' involvement in medicines development is getting more and more established in both academic and industrial settings, with numerous publications showing the positive impact it might have (9, 10). The European Patient Academy of Therapeutic Innovation (EUPATI) launched by the Innovative Medicines Initiative (IMI) is an example of a successful consortium that promotes patient participation across various stages in the drug development lifecycle (11). This is essential to ensure that patients' expectations from novel therapeutics are at the center of trials' objectives. Now is the time to apply this principle in the search for novel treatments and vaccines against COVID-19. Facing the tsunami of informations becoming available every day about SARS-CoV-2 and COVID-19, patients' input is also essential to determine what type of information is relevant and how it should be communicated (12).

In order to take advantage of the current window of opportunity for patient's involvement in the fight against COVID-19 pandemic, one should consider the various phases of the patient journey, from the pre-symptomatic phase up to recovery and beyond. The multisystemic consequences of SARS-CoV-2 infection complexifies patient stratification and delineation of patient preferences. Furthermore, patient reported outcomes are essential to assess the impact of therapeutic interventions on the long-term consequences of COVID-19 (13). It is therefore important that patient organizations are involved early on in the design of clinical trials.

Finally, as compared to medical interventions for other diseases, one should keep in mind that during a pandemic every citizen can become a patient from 1 day to the other. This has impact on both clinical trial recruitments and behavioral interventions to reduce the spread of the disease, since healthy people need stronger incentives as compared to patients. This is especially important to ensure adequate vaccine coverage in an era in which anti-vaxx movements are very active and vaccine hesitancy is prevalent. Fostering health literacy in the wider public should therefore closely be associated with patient empowerment in the collective fight against COVID-19.

## Data Availability Statement

The original contributions presented in the study are included in the article/supplementary material, further inquiries can be directed to the corresponding author/s.

## Author Contributions

All authors listed have made a substantial, direct and intellectual contribution to the work, and approved it for publication.

## Conflict of Interest

The authors declare that the research was conducted in the absence of any commercial or financial relationships that could be construed as a potential conflict of interest.
